# Epigallocatechin Gallate Effectively Affects Senescence and Anti-SASP via *SIRT3* in 3T3-L1 Preadipocytes in Comparison with Other Bioactive Substances

**DOI:** 10.1155/2020/4793125

**Published:** 2020-10-21

**Authors:** Stephanie Lilja, Julia Oldenburg, Angelika Pointner, Laura Dewald, Mariam Lerch, Berit Hippe, Olivier Switzeny, Alexander Haslberger

**Affiliations:** ^1^Department of Nutritional Sciences, University of Vienna, 1090 Vienna, Austria; ^2^HealthBioCare GmbH Nußdorferstraße 67, 1090 Wien, Austria

## Abstract

**Aim:**

We investigated different bioactive compounds including epigallocatechin gallate (EGCG), anthocyanidin, resveratrol, phloretin, spermidine, butyrate, and *β*-hydroxybutyrate with regard to their effect on *SIRT3* via *NRF2* and modulation of the proinflammatory senescence-associated secretory phenotype (SASP) in senescence induced 3T3-L1 preadipocytes.

**Methods:**

For induction of senescence, 3T3-L1 preadipocytes were incubated with bromodeoxyuridine (BrdU) for 8 days. Cell cycle inhibition was observed, and *β*-galactosidase activity was measured. After BrdU treatment, cells were treated with different bioactive compounds in various concentrations for 96 h. ELISA was used for determining proinflammatory cytokine IL6 in SASP cells.

**Results:**

*CDKN1a* increased significantly after BrdU incubation compared to untreated control (*p* < 0.01). All secondary plant ingredients used for treatment, but not anthocyanidin 50 *μ*M, decrease *CDKN1a* expression (*p* < 0.05), whereas most endogenous substances did not attenuate *CDKN1a*. IL6 secretion positively correlated with *CDKN1a* (*p* < 0.01), whereas EGCG could diminish both, IL6 and *CDKN1a* with the strongest effect (*p* < 0.01). Although *NRF2* positively correlated with *SIRT3* activation (*p* < 0.05), only resveratrol (*p* < 0.01) and anthocyanidin (*p* < 0.05) could activate *NRF2* significantly. Solely anthocyanidin 50 *μ*M (*p* < 0.05) and 100 *μ*M (*p* < 0.01) and EGCG 50 *μ*M (*p* < 0.01) could increase *SIRT3* expression. Activation of *SIRT3* with EGCG correlated with lowered IL6 secretion significantly (*p* < 0.05) but not with anthocyanidin.

**Conclusion:**

Accumulation of senescent cells in adipose tissue plays an important role in obesity and age-related diseases. *SIRT3*, located in the mitochondria, can regulate ROS via different pathways. Thus, targeting *SIRT3* activating compounds such as EGCG may delay senescence of cells and senescence induced inflammatory processes.

## 1. Introduction

In the past decade, senescence cells have emerged as possible contributors to the pathogenesis of many age-related diseases, potentially caused by cytokines released due to metabolic stress [1, 2]. Cells gradually lose their division potential under normal culture conditions, also called replicative senescence. They may, however, also enter premature senescence, a similar state, induced by various stimuli, including oncogene activity, oxidative stress, and DNA damage [1]. Depending on the factors involved, different pathways are activated resulting in the activation of *p53* and its downstream target *CDKN1a* [2]. Activation of cell cycle inhibitors, like *CDKN1a*, is considered as a hallmark of senescence [3]. The central purpose of cells undergoing senescence is to eliminate damaged cells by the immune system. Yet, if the clearance is impaired, it can lead to accumulation and tissue dysfunction. Senescence cells remain metabolically active. If they persist, they consequently alter their microenvironment and nearby cells by implementing a complex proinflammatory response, and thus acquiring the SASP. The SASP is mediated by transcription factor nuclear factor *κ*B (NF-*κ*B) and CCAAT/enhancer binding protein-*β* (CEBP*β*), including the secretion of proinflammatory cytokines (IL6 and TNFa), chemokines, macrophage inflammatory proteins (MIP) as well as transforming growth factors (TGF*β*). Latter upregulates p21 through the SMAD complex [2]. Another stimulus which provokes the development of aging phenotypes is mitochondrial dysfunction, resulting in cellular senescence in vitro and in vivo [4]. Mitochondria oxidize NADH to NAD+, which declines with aging. NADH is generated by the TCA cycle, but cytosolic NAD+/NADH pool is also used for oxidation to NAD+. A lower NAD+/NADH ratio induces senescence and has been shown to be associated with aging [4]. Further, mitochondrial dysfunction impairs metabolism and redox homeostasis, which is related to several chronic diseases, like diabetes type 2, obesity, metabolic syndrome, and development of age- and diabetes-dependent liver steatosis [5, 6]. The largest internal organ in humans, the fat tissue, is strongly involved in longevity and age-related metabolic dysfunctions. Besides its main role of storing highly reactive fatty acids as triglyceride in lipid droplets, fat is important for several essential physiological processes including immune function, thermoregulation, mechanical protection, and tissue regeneration. However, in doing this, the adipose tissue is a highly active endocrine system and secretes hormones such as leptin, adiponectin, growth factors, and cytokines like IL6 [7]. Throughout life, fat distribution and its function change depending on genetic and epigenetic disposition as well as lifestyle factors [4, 7, 8]. Towards middle age, the proportion of fat tissue begins to decrease and further declines in old age. Preadipocytes are related to macrophages and give rise to new adipocytes. These fat cell progenitors are 10fold more abundant in obese subjects, but dysdifferentiate within aging [7].

Both aging and obesity are associated with a chronic, low-grade inflammation, potentially fueling the development of diabetes, hypertension, cancer, cognitive dysfunction, atherosclerosis, and thus diminishing health span [7, 8]. When it comes to aging, a class of NAD+ dependent histone deacetylases (HDACs), called sirtuins, has been frequently mentioned as they are involved in the regulation of transcription, apoptosis, stress resistance and furthermore act as caloric energy sensor [9, 10]. HDACs are grouped in four classes, which all have in common to regulate gene expression by removing acetyl groups in histones [11]. HDAC class III includes seven members of sirtuins (SIRT1-7), identified in mammals, which are localized in different cellular compartments [9, 11]. SIRT1, 2, 6, and 7 can be found in the nucleus; SIRT1 and SIRT2 in cytoplasm; and SIRT3, 4, and 5 are localized in the mitochondria [9]. Increasing data support their role in modulation of cellular senescence and lifespan in different animal models [5]. SIRT3 regulates several aspects of mitochondrial function and is a promising candidate to diminish oxidative stress, thus inflammation and age-related diseases [9]. Interestingly, it has been demonstrated that activation of nuclear factor erythroid 2–related factor 2 (*NRF2*) induces *SIRT3* gene expression via antioxidant response element (ARE) in its enhancer region [12]. *NRF2* is a transcription factor important as the first cellular defense against oxidative stress [6, 12]. Usually sequestered together with Kelch-like ECH-associated protein 1 (Keap1) in the cytoplasm, *NRF2* dissociates during oxidative stress and translocates to the nucleus and furthermore induces transcription of genes with ARE in their regulatory region [12]. *NRF2* and *SIRT3* have been discussed as therapeutic targets to reduce senescence in adipose tissue and thus decreasing chronic low-grade inflammation and prevent different disorders [5, 12–14].

In the last decade, a large group of bioactive compounds, including flavonoids, like EGCG and anthocyanidins as well as the stilbene resveratrol, have been identified to target senescence via multiple pathways [15]. Many effects are due to the modulation of epigenetic mechanisms [5, 8, 14]. A large group of bioactive compounds have been identified to activate *SIRTs* and *NRF2* [5, 12, 13, 16]. In general, senescence of cells can be eliminated by the activation of the immune system or targeting individuals SASP factors, which might prevent the paracrine spread of senescence, thus inflammation [8]. Another approach is to identify compounds with senolytic effects inducing apoptosis in senescent cells and prevent their accumulation [17]. Especially, secondary plant ingredients like polyphenols, and their synergistic effect when combined, are highly interesting compounds with anti-SASP activity. Furthermore, their anti-inflammatory and antioxidative capacity might be useful in the treatment and prevention of metabolic syndrome [15, 16, 18]. Some bioactive compounds are known to activate sirtuins, thus having beneficial effects for human health [11]. Also, caloric restriction and ketogenic diet have become more popular for their anti-inflammatory effect and health benefits. Caloric restriction is linked to higher levels of ketone bodies such as *β*-hydroxybutyrate (BHB), which is produced by ketogenesis in liver mitochondria and released into the bloodstream as energy fuel. The production of ketone bodies is mediated by *SIRT3* and may prevent metabolic dysfunctions, like insulin resistance and obesity by activating antioxidative defenses [19]. Butyrate, another short-chain fatty acid produced by the gut microbiota, can inhibit HDAC class 1 and thus maintain homeostasis and oxidative status [20]. A further promising molecule in this context is spermidine, a polyamine which stabilizes DNA and RNA, has antioxidative capacities, and ables to modulate various enzyme functions. In mammals, polyamine levels strongly depend on their nutritional supply as well as its synthesis by the intestinal microbiota. However, polyamine proportion declines within age [21].

The aim of this study was to characterize the effects of different bioactive compounds on senescence status and gene expression of senescence-induced cells. As polyphenols constitute a promising substance group in this context, we investigated EGCG, anthocyanidin, resveratrol, and phloretin, but also spermidine, butyrate, and BHB with regard to their effect on *SIRT3* via *NRF2*, modulation of senescence, and SASP in senescence-induced 3T3-L1 preadipocytes. Senescence was induced with a sublethal dose of BrdU, which is widely used to measure DNA synthesis in proliferating cells and has been known to alter growth and phenotype of different cells. BrdU as a 5-halogenated thymidine analogue is incorporated into the DNA which can result in DNA hypermethylation, mutations, chromatid breaks, and other lesions and induce a senescence-like phenomenon in every type of mammalian cell [22, 23].

To assess potential synergistic effects, cells were treated with these substances only, as well as with a mixture of EGCG, resveratrol, and spermidine.

## 2. Material and Methods

### 2.1. Cell Culture

3T3-L1 preadipocytes were sponsored by the Department of Nutritional science Vienna. Cells were cultured as a monolayer in Dulbecco's modified Eagle medium (DMEM) high glucose (4.5 g/l) containing L-glutamine, 5% penicillin/streptomycin and 10% fetal calf serum at 37°C in a humidified atmosphere of 95% air and 5% CO2. Studies were performed in the passage numbers 3 to 5. Cells were passaged before reaching confluency using 1x PBS and Accutase solution (all substances from Merck, Germany). This cell line was chosen because senescence in preadipocytes impairs their function and the cytokines released in senescent cells can be spread to nonsenescent neighbored preadipocytes. Moreover, the release of cytokines is highly secreted in preadipocytes of older rats and can impair the recruitment of immune cells [15].

### 2.2. Substances

All substances were chosen from literature based on their impact to modulate senescence, their antioxidative properties, or their impact on health. Polyphenols and the polyamine used for this study were EGCG, anthocyanidin, resveratrol, and spermidine sponsored by System-Biologie AG (Switzerland). As secondary plant ingredients are reported to have potential synergistic effects, we tried a mixture of EGCG 40%, resveratrol 40%, and spermidine 20%. Used plant compounds are extracts of different plants. EGCG was extracted from the leaf of Camellia sinensis O. Kuntze; anthocyanidin was obtained from blueberries, with the major component of cyanidin. Resveratrol was extracted from the roots of Polygonum cuspidatum sieb. et Zucc and spermidine from wheat germ. Butyrate (B5887), *β*-hydroxybutyrate (54965), phloretin (P7912), and roxithromycin (R4394) were purchased from Merck (Germany). After testing for cytotoxicity, we used the following concentrations for experiments: EGCG 50 *μ*M and 100 *μ*M, anthocyanidin 50 *μ*M and 100 *μ*M, phloretin 50 *μ*M and 100 *μ*M, resveratrol 10 *μ*M and 15 *μ*M, EGCG-resveratrol-spermidine mix 20 *μ*M and 30 *μ*M, spermidine 5 *μ*M and 10 *μ*M, butyrate 2.5 mM and 5 mM, and BHB 4 mM and 10 mM. Roxithromycin has antisenescence properties after BrdU treatment [23] and was used as positive control.

### 2.3. Cell Proliferation

The proliferative potential of cells during treatment was assessed by the 3-(4,5-dimethylthiazol-2-yl)-2,5-diphenyltetrazolium bromide (MTT; Merck) assay. Cells were seeded in 96-well microplates at a density of 5000 cells/well for the 24 h assay, 3000 cells/well for 48 h, and 1000 cells/wells for the 72 h assay. They were cultured for two days. Different densities and incubation times were only used for assessment for cytotoxicity to see potential cytotoxicity in confluent and nonconfluent cultures. Cells were treated with several different concentrations for each substance and each concentration was done in triplicates. After the respective treatment times, the media of the plates was discarded, and MTT was added to the wells together with DMEM high glucose without phenol red (Merck). This was followed by another incubation for 4 h at 37°C in a humidified CO2 atmosphere. The media was again discarded and 100 *μ*l of acid isopropanol (0.1 N HCl in anhydrous isopropanol) were pipetted to each well to solubilize formazan precipitates. The absorbance of purple formazan was measured at a wavelength of 540 nm using a FLUOstar OPTIMA microplate reader (BMG Labtech). Each compound was assessed for cytotoxicity with several different concentrations and different timepoints. Proliferation rate and cytotoxicity were calculated relatively to the proliferating control cells, and concentrations for further experiments were determined by IC50 and listed in the chapter substances.

### 2.4. Senescence Induction and Treatments

**Figure 1:**
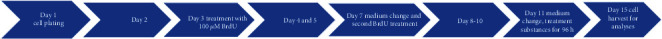
The experimental design outlined per day. Timeline for cell plating and senescence induction and incubation with different substances are addressed in this figure.

Cells were plated in 24-well plates at a density of 3000 cells/cm^2^. Around 80% confluency, cells were exposed to 100 *μ*M BrdU for 8 days, including one medium change containing BrdU after 4 days. The concentration of BrdU and incubation length of all substances was determined based on the literature and proliferation assay results [23]. After final treatment with BrdU, cells were washed with PBS and kept for additional 96 h in fresh DMEM containing different substances, each in different concentrations. For testing anti-SASP effects all concentrations and substance combinations as mentioned above were applied for experiments. This set of experiments was done for *β*-galactosidase staining, ELISA and gene expression analysis, and for each analysis done in triplicates and different timepoints. By day 8 of exposure to BrdU, treated cells acquire the senescence-like phenotype. The most widely used assay for senescence detection is the senescence-associated *β*-galactosidase activity at pH 6.0. The activity is based on the increased lysosomal content and reflects the increased autophagy as well as the enlarged lysosomal compartments in the cells. Cells were plated in 6-well culture plates at a density of 3000 cells/cm^2^. Cellular senescence was identified using the *β*-galactosidase assay (Biovision). At the end of the experiment, medium was aspirated from the cells, washed with PBS, and then fixed with fixation solution. Cells were washed again, stained, and incubated overnight at 37°C in absence of light and CO_2_. Next day, cells were observed under microscope (Leitz LG91 Diavert wetzlar Germany) for the appearance of blue color as a marker of senescence associated *β*-gal activity. Several images were taken, and cells were manually counted. The experimental design is outlined in Figure 1.

### 2.5. ELISA

After respective treatments, the supernatants of cells were collected for detecting IL6 levels using a sandwich ELISA kit (Mouse IL-6 ELISA Kit Invitrogen by Thermo Fisher Scientific).

### 2.6. RT2-PCR

RNA was extracted using the MagMAX™ *mir*Vana™ Total RNA Isolation Kit via King Fisher Duo Prime (ThermoFisher Sientific). Up to 1 *μ*g template RNA was used for reverse transcriptase and cDNA amplification using TagMan Reverse Transcription Reagents (ThermoFisher Sientific). Real-time PCR was performed using *GAPDH* as housekeeping gene and *SIRT3*, *CDKN1a* and *NRF2* as genes of interest using PCR condition, primer assay, and mastermix according to the manufacturer's protocol (all ThermoFisher Scientific). For comparison of runs, an untreated control was used on every plate. Relative expression was calculated using *^ΔΔ^*ct method and expressed as 2^-*ΔΔ*Ct^ using the Gene Expression Software.

### 2.7. Statistical Analyses

Data was analyzed using the GraphPad Prism (Version 6) software and data are presented as mean ± standard deviation (SD). Each experiment was done at least three times. Statistical differences between control and experimental groups were determined using one-way ANOVA (*p* < 0.01) with Dunnett's post hoc test.

## 3. Results

### 3.1. BrdU Induces Senescence-Like Morphology in 3T3-L1 Cells

**Figure 2:**
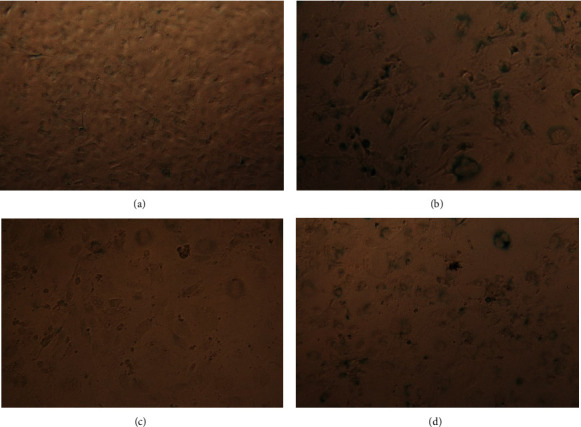
Beta galactosidase activity staining (blue) in 3T3-L1 preadipocytes. Untreated confluent 3T3-L1 preadipocytes show minor amount of senescence cells (a). After BrdU treatment for 8 days, cells show a typical senescence phenotype and increased beta galactosidase activity, which is indicated by blue staining (b). After induction of senescence with BrdU for 8 days, following a treatment with roxithromycin 100 *μ*M for 96 h beta gallactosidase activity decreased resulting in significant less blue stained cells (c). EGCG 100 *μ*M treatment for 96 h after 8days of BrdU treatment could decrease *β*-gal activity but not in the extend as roxithromycin (d).

Cells treated with BrdU developed a typical senescence like morphology. *β*-gal staining displayed large, flat, multinucleated, and enlarged nuclei compared to the control (Figure 2(b)). Furthermore, BrdU inhibited proliferation of 3T3-L1 cells.

### 3.2. Polyphenols Reduce Senescence Genotype Significantly

**Figure 3:**
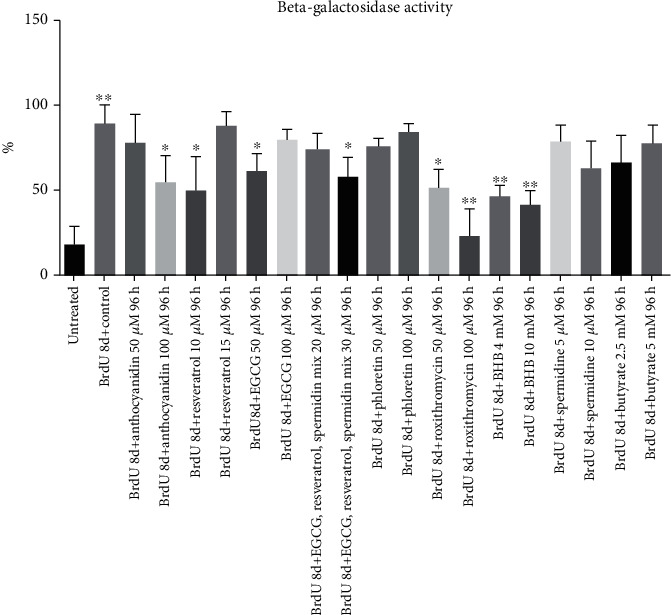
Percentage of *β*-galactosidase activity of all substances and concentrations in response to BrdU control. Beta-gal activity was significantly enhanced in BrdU cells compared to untreated cells (*p* < 0.01). Anthocyanidin, EGCG, resveratrol, EGCG-resveratrol-spermidine mix, BHB, and roxithromycin could change senescence phenotype and diminish beta-gal activity significantly (^∗^*p* < 0.05; ^∗∗^*p* < 0.01). The results were expressed as mean ± SD. Statistical significance between compounds and concentrations to the control was determined by one-way ANOVA with Dunnett's post hoc test.

**Figure 4:**
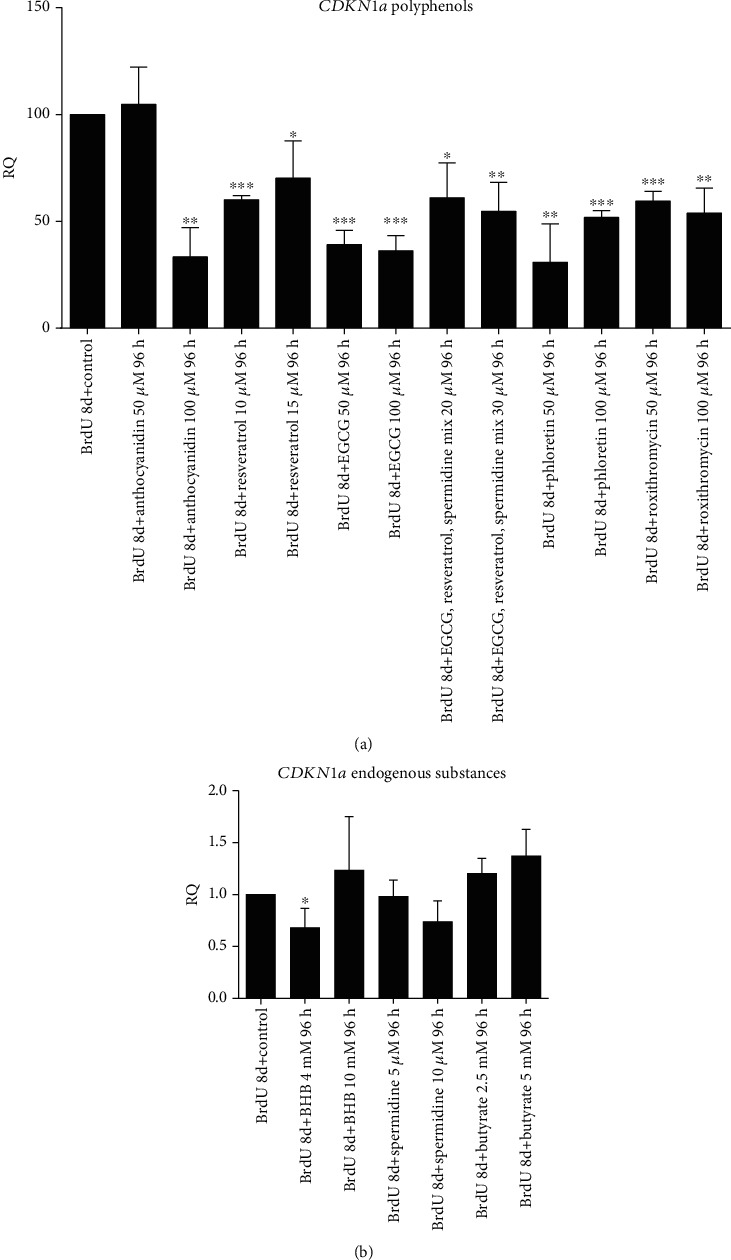
RQ value of *CDKN1a* mRNA expression. Cells incubated with BrdU showed an increase in gene expression, thus cell cycle arrest. Subsequent treatment with different substances showed a decrease of *CDKN1a* expression with secondary plant ingredients (a), but for endogenous substances this result could only be reached for 4 mM BHB (b). Statistical significance was defined ^∗^*p* < 0.05; ^∗∗^*p* < 0.01; ^∗∗∗∗^*p* < 0.001. The results were expressed as mean ± SD. Statistical significance between compounds and concentrations to the control was determined by one-way ANOVA with Dunnett's post hoc test.

Assessment of *CDKN1a* gene expression of treated cells compared to untreated control was applied for verification of *β*-gal activity results (*p* < 0.01). Most substances could decrease *β*-gal activity, whereas not all changes were significant (Figure 3). Besides cells treated with 50 *μ*M anthocyanidin, all other polyphenols reduced the senescence genotype significantly. BHB in a physiological concentration of 4 mM, which can be reached after a 5 days fasting period [24], reduced *CDKN1a* gene expression significantly (*p* < 0.05). Higher concentrations of BHB, representing a long-time fasting intervention, attenuated gene expression of this cell cycle inhibitor. For the other endogenous substances, no significant changes could be observed (Figure 4).

### 3.3. Polyphenols except Resveratrol Decrease IL6 Secretion in BrdU-Treated Cells

**Figure 5:**
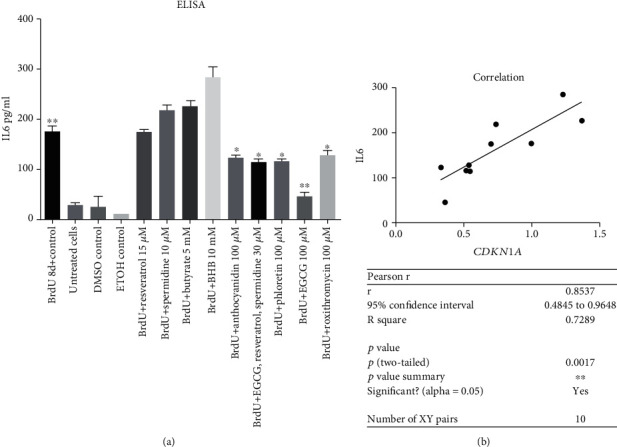
IL6 levels pg/ml measured with ELISA in cells treated with BrdU and different substances, using the highest concentration. Compared to untreated cells IL6 secretion increased significantly (*p* = 0.002). Regarding BrdU control, most secondary plant ingredients besides resveratrol could decrease IL6 secretion significantly. Statistical significance was defined ^∗^*p* < 0.05; ^∗∗^*p* < 0.002. The results were expressed as mean ± SD. Statistical significance between compounds and concentrations to the control was determined by one-way ANOVA with Dunnett's post hoc test (a). Pearson's correlation analysis showed a significant correlation of *CDKN1a* expression with IL6 secretion including all substances (^∗∗^*p* < 0.002) (b).

To assess the SASP state of treated cells, secretion of proinflammatory cytokine IL6 was assessed with ELISA. Analysis included only the highest concentration of substances. We could observe, that IL6 secretion increased strongly in BrdU-treated cells (*p* < 0.002). Except for resveratrol, all polyphenols inversed cytokine secretion significantly, with EGCG 100 *μ*M showing the strongest effect (*p* < 0.002) (Figure 5(a)). Furthermore, IL6 secretion positively correlates with *CDKN1a* expression (*p* < 0.002) (Figure 5(b)).

### 3.4. *NRF2* Gene Expression Was Higher in BrdU-Treated Cells

**Figure 6:**
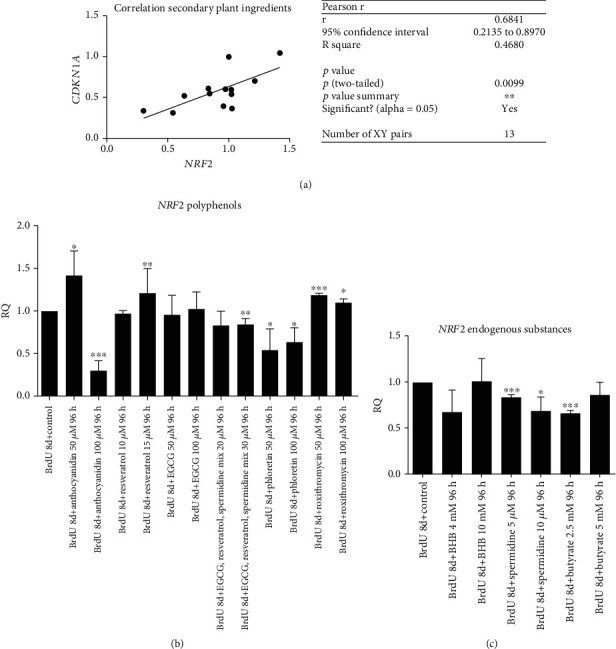
Pearson's correlation between *CDKN1a* and *NRF2*. Cell cycle arrest thus increase in *CDKN1a* positively correlates with *NRF2* expression (*p* < 0.01) (a). Not all secondary plant ingredients are *NRF2* activators. Relative quantification values of *NRF2* comparing all treatments regarding BrdU control (b+c). Anthocyanidin 50 *μ*M, resveratrol 15 *μ*M, and both roxithromycin concentrations increased *NRF2* antioxidative defense pathway significantly (b). EGCG, resveratrol, spermidine mix 30 *μ*M, both phloretin concentrations, but also endogenous substances, like spermidine and butyrate diminished *NRF2* assuming lower ROS (b+c). Statistical significance was defined ^∗^*p* < 0.05; ^∗∗^*p* < 0.01; ^∗∗∗^*p* < 0.005; ^∗∗∗∗^*p* < 0.001. The results were expressed as mean ± SD. Statistical significance between compounds and concentrations to the control was determined by one-way ANOVA with Dunnett's post hoc test.

BrdU-treated cells showed a significant increase in *NRF2* gene expression compared to untreated control cells (*p* < 0.05). *NRF2* gene expression of polyphenol treated cells positively correlates with senescence induction (*p* < 0.01) (Figure 6(a)). Conversely, cells treated with endogenous substances as well as treatments with 100 *μ*M anthocyanidin and 50 *μ*M or 100 *μ*M phloretin inhibited *NRF2* gene expression (Figure 6(b)). Although resveratrol and EGCG are known as a *NRF2*-activating polyphenol, only resveratrol 15 *μ*M (*p* < 0.01) and anthocyanidin 50 *μ*M (*p* < 0.05) could activate *NRF2* significantly.

### 3.5. *SIRT3* Gene Expression Could Be Elevated by Anthocyanidin and EGCG

**Figure 7:**
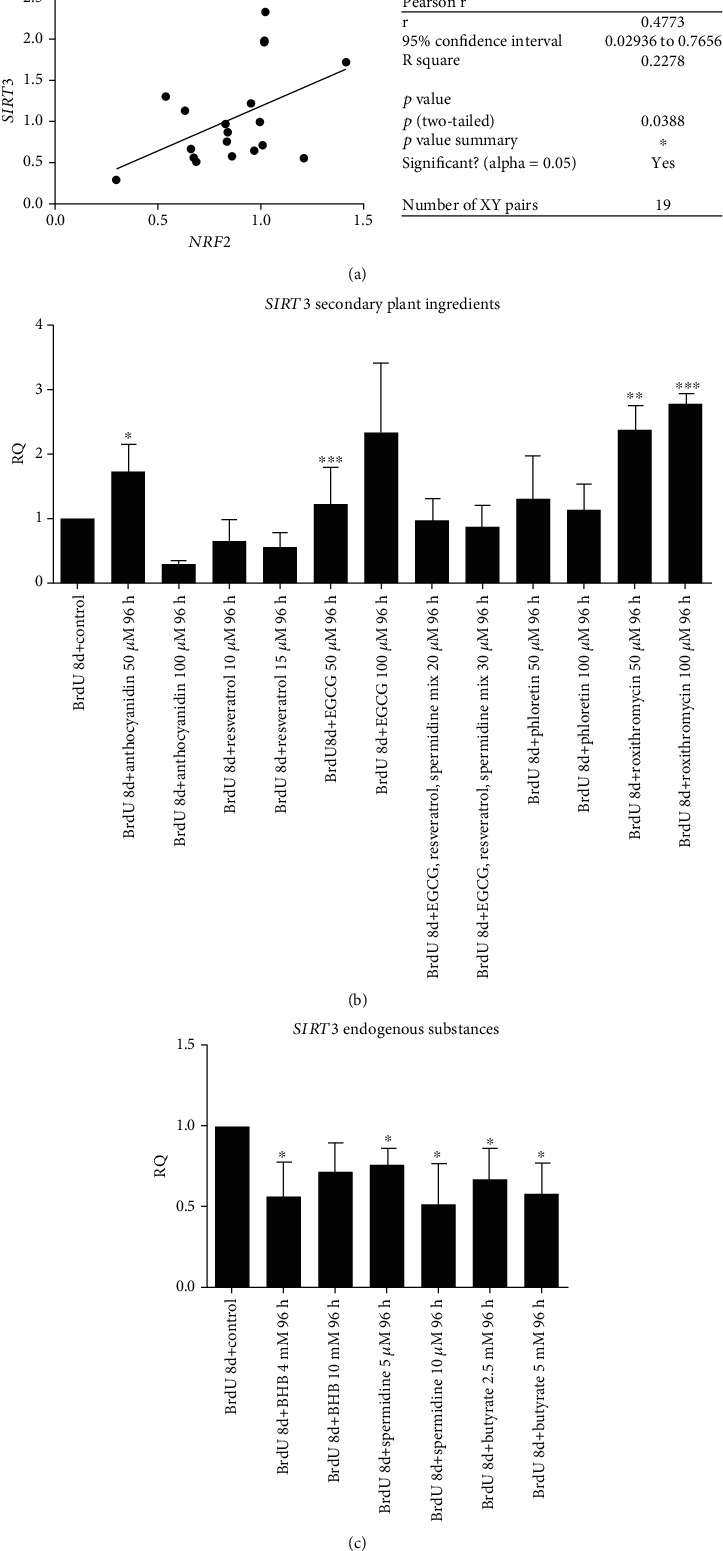
Positive Pearson's correlation between *NRF2* and *SIRT3* expression of all substances (a). Relative quantification values of *NRF2* comparing all treatments regarding BrdU control (b+c). Significant activation of *SIRT3* can be seen with anthocyanidin and EGCG both at a concentration of 50 *μ*M. EGCG 100 *μ*M increased *SIRT3* expression but not significant (b). Both concentrations of roxithromycin increased *SIRT3* expression. The other secondary pant ingredients did either not influence *SIRT3* expression or like the endogenous substances ameliorate *SIRT3* (c). Statistical significance was defined ^∗^*p* < 0.05; ^∗∗^*p* < 0.01; ^∗∗∗^*p* < 0.005; ^∗∗∗∗^*p* < 0.001. The results were expressed as mean ± SD. Statistical significance between compounds and concentrations to the control was determined by one-way ANOVA with Dunnett's post hoc test.

**Figure 8:**
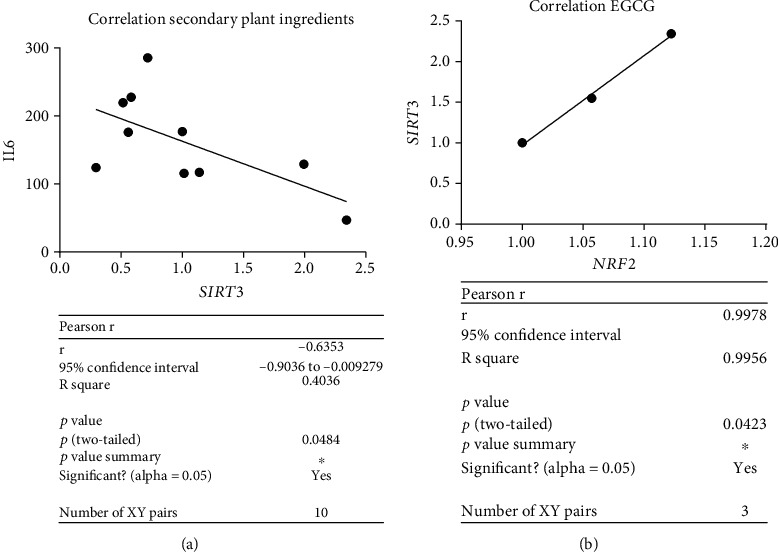
Pearson's correlation IL6 levels pg/ml and *SIRT3* gene expression RQ regarding treatment with secondary plant ingredients. *SIRT3* activation diminished IL6 secretion in senescent cells, thus ameliorating inflammation regarding secondary plant ingredients but not endogenous substances (*p* < 0.05) (a). Positive Pearson's correlation *SIRT3* and *NRF2* of EGCG in a dose dependent manner (0 *μ*M, 50 *μ*M, and 100 *μ*M) (*p* < 0.05) (b).

No correlation of *SIRT3* activation could be seen by increased *CDKN1a* gene expression. Our results show positive correlation of *SIRT3* expression and *NRF2* (*p* < 0.05) (Figure 7(a)) and reduced IL6 levels (*p* < 0.05) (Figure 8(a)). Latter could only be generated for the secondary plant ingredients. Only anthocyanidin 50 *μ*M and EGCG 50 *μ*M increased *SIRT3* significantly compared to roxithromycin 50 *μ*M and 100 *μ*M (Figure 7(b)). Although EGCG did not significantly stimulate *NRF2* expression, there is a dose-dependent correlation of *NRF2* and *SIRT3* activation (*p* < 0.05), which we could not see for anthocyanidin (Figure 8(b)).

## 4. Discussion

Senescence cells can have both positive and adverse effects, depending on the disease or tissue. In cancer or liver fibrosis, senescence can be beneficial, thus restricting tumor progression and fibrosis, whereas in metabolic disorders it may be detrimental by contributing to the disease [2]. Reaching the threshold of storage capacity in adipocytes by caloric overload triggers a stress response and macrophage recruitments [2]. Oxidative stress is of great interest to the study of obesity and its pathologies, like metabolic syndrome and diabetes, thus diminishing health span [25]. 3T3-L1 cells were chosen because the release of cytokines from preadipocytes influences the function of fat tissue, further the recruitment of immune and inflammatory cells leading to inflammatory states and pathological complications [15]. Exposure to a high fat diet leads to oxidative stress in a variety of tissues within the body. For this reason, studying senescence and oxidative stress and their effects on adipose tissue as well as the anti-SASP effects of different secondary plant ingredients and endogenous substances is of critical importance. *NRF2* and *SIRT3* play essential roles in the regulation of antioxidant defense [25]. Mitochondrial DNA damage is the molecular basis of cell senescence, and mitochondrial oxidative stress accumulation is a major factor to determine age related diseases and lifespan [13, 26]. Like Ozsvari et al., we used BrdU as a senescence inducer in 3T3-L1 preadipocytes to mimic obesity induced inflammation. Consistent with his study, we observed that an application of sublethal BrdU concentrations could activate the senescence program in cells, including enlarged cell sizes, expression of SA-*β*-Gal, increased *CDKN1a* expression and development of SASP, by measuring IL6 levels [23]. Proinflammatory cytokines including IL6 are detrimental for the nearby cells by spreading inflammation leading to a disturbed tissue function [17]. BrdU activates cell cycle inhibitor *CDKN1a* and suppresses cell proliferation. In recent years, secondary plant ingredients, such as EGCG, resveratrol, and other flavonoids, have been investigated regarding their senolytic and antiaging properties [5]. In our study, all secondary plant ingredients but also BHB at a lower concentration diminished *CDKN1a* expression and improved cell proliferation, but only polyphenols reduced the levels of IL6 secretion significantly. IL6 is a major cytokine, secreted in the SASP. Polyphenols have anti-inflammatory and antibiotic properties and may in addition activate the transcription factor *NRF2*. *NRF2* plays a key role in cellular protection against oxidative stress and inflammation [27]. In the study of Liu et al., cells were treated with different substances to induce genotoxic stress. In combination with BHB *p53* and its downstream target *CDKN1a* was deceased, induced by *β*-hydroxybutyrylation which is a novel histone posttranslational modification [28]. Spermidine and butyrate are known to reduce inflammation by targeting NF-*κ*B, G-protein–coupled receptors, autophagy, or inhibiting HDAC class1 [29, 30]. In addition, the expression of many SASP components such as IL8 or IL6 are regulated by the activity of the transcription factor NF-*κ*B, responsible for the development of inflammation [31]. In our study, we could not observe any significant anti-inflammatory effects of spermidine and butyrate. In this regard, the use of BrdU as strong genotoxic agent could alleviate potential beneficial effects of compounds. Further studies could include lower BrdU concentrations to assess anti-inflammatory effects. A chronic SASP has been associated with the spread of senescence and a high proinflammatory status, consequently contributing to a faster aging process, which can be also found in obesity and diabetes type 2 [5]. Therefore, a targeted modulation of SASP and senescence may constitute a powerful tool to increase health span. Polyphenols are auspicious compounds with multiple beneficial health effects including antioxidative and anti-inflammatory properties [5]. Their anti-SASP properties are of special interest. Their ability to activate ARE and *NRF2* is determined by structural features. EGCG, phloretin, and related substances have the potential to induce *NRF2*, but only anthocyanidin and resveratrol could significantly increase *NRF2* in our study [16]. *NRF2* is a stress responsive transcription factor balancing redox homeostasis by activating genes that encode cytoprotective, antioxidant, and phase II detoxifying enzymes [16, 32]. Several natural compounds have been identified as electrophilic *NRF2* inducers, like resveratrol, as we could also demonstrate in our results [33]. The stimulation of *NRF2* by exogenous substances (e.g., sulforaphane) leads to a decreased translocation of NF-*κ*B to the nucleus, consistent with a lower DNA binding capacity and diminished proinflammatory action [34]. Nevertheless, *NRF2* can also interact with *CDKN1a*, indicating that *NRF2* is upregulated in cellular senescence [16], which goes in line with our observations, in particular after treatment with 50 *μ*M anthocyanidin. When exposed to electrophiles or oxidative stress, cysteine residues of Keap1 are modified to prevent it from targeting *NRF2* for proteasome degradation, resulting in rapid accumulation of *NRF2*. *CDKN1a* can stimulate or promote *NRF2* activation while *p53* or *p65* decrease *NRF2* transcription [34]. *CDKN1a* protects cells against oxidative stress through upregulation of the NRF2 signaling pathway and may be the first defense mechanism used to reduce reactive oxygen species (ROS) under low stress conditions [16]. Mitochondrial ROS production induces different cell signals mediated by protein phosphorylation, NO synthase, and *NRF2*, which downregulate ROS by their feedback [35]. Apoptosis requires ROS accumulation, and consistent with that apoptosis is induced at high oxidative stress. *NRF2* antioxidant response pathway must possibly be suppressed in order to induce apoptosis, which might mean that anthocyanidin (in higher concentration) and phloretin as well as some of the endogenous substances used in our study act as a senolytic [16]. Kumar et al. investigated the effect of EGCG on senescent cells and observed a senolytic effect by diminished *NRF2* expression and inhibiting *Bcl2* [17]. In contrast, different studies summarize antiapoptotic effect of natural and synthetic compounds due to increased *NRF2* expression and ameliorating *Bcl2* expression [5, 16, 36]. In our study, endogenous substances, like spermidine, BHB, and butyrate, did neither increase *NRF2* nor decrease *CDNK1a* and IL6 levels. Nevertheless, most secondary plant ingredients, including the EGCG-resveratrol-spermidine mix, diminish *CDKN1a* and IL6 levels, although only anthocyanidin and resveratrol activated *NRF2* significantly. *NRF2* can be activated both dependently and independently from Keap1. Latter activation is either due to oxidative stress or other *NRF2* activators. Following this, *NRF2* translocate from the cytoplasm into the nucleus and binds to ARE of different genes [32, 37]. Butyrate, but also BHB, has been shown to be potent *NRF2* activators in different studies [38–40]. In contrast to our results, Kwak et al. demonstrate spermidine as a phase 2 enzyme inducer due to NRF2-ARE pathway activation [37]. In addition, recent studies indicate that *NRF2* binds directly to the *SIRT3* promoter, which leads to an increase in its expression [41, 42]. Although resveratrol increased *NRF2* expression in our study, *NRF2* further did neither stimulate *SIRT3* expression nor IL6 secretion was reduced. One possible explanation could be that resveratrol is not able to diminish genotoxic stress, caused by BrdU while inducing senescence. The most consistent data was generated for roxithromycin, a drug described for their antisenescence properties [23]. Roxithromycin attenuated senescence status regarding *CDKN1a* and IL6 secretion and increased *SIRT3* expression via *NRF2*. Compared to the other treatment compounds, EGCG showed similar characteristics in terms of results in gene expression. Although EGCG did not increase *NRF2* significantly in our study, it activated *SIRT*3 leading to reduced proinflammatory cytokine secretion and decreased *CDKN1a* expression. EGCG is known for its beneficial effects on human health, preventing inflammatory diseases attributed to its antioxidative, anti-inflammatory, radical scavenging, metal chelating, and anticarcinogenic properties [43]. Although IL6 levels decreased in senescent cells after most polyphenol treatments, our results suggest that this is not only by reason of *NRF2* activation but rather, due to a direct activation of *SIRT3*. However, *NRF2* activation is depending on the chemical structure of the bioactive compounds [14, 32]. Interestingly, *SIRT*3 activity can reduce ROS levels by directly modulating key antioxidant enzymes, thereby acting as a shield against oxidative damage. *SIRT*3 deacetylates manganese superoxide dismutase (MnSOD) via FoxO3a in mitochondria and increases the ability to diminish ROS. Further, it activates isocitrate dehydrogenase 2 (IDH2) which produces NADPH needed for generating glutathione (GSH). Together with FoxO3a, *SIRT*3 upregulates all 13 mitochondrial encoded genes, resulting in an increase in mitochondrial respiration and biogenesis [19, 41, 44]. Thus, *SIRT*3 protects mitochondrial function, including ATP generation and mitochondrial membrane potential. However, studies show a *SIRT*3 deficiency does not diminish increased ROS [41, 45]. Furthermore, reduced *SIRT3* expression is associated with cell aging and downregulated in metabolic syndrome, hyperlipidemia, diabetes, and smoking, thus related with human longevity [35, 46]. Dysfunction in the largest organ in humans, the fat tissue with its central role in metabolism is related to the onset of many age-related diseases [7]. Targeting compounds able to activate *SIRT*3 in senescent; proinflammatory cells can have profound clinical consequences.

## 5. Limitations

Like in various other in vitro studies, high concentrations of substances over physiological levels were used. However, in vivo studies would not achieve the concentrations of some substances. Moreover, it would be interesting to investigate not only anti-SASP effects of these substances but also compare their senolytic effect, including markers, like *Blc2* and a comparison of preadipocytes and mature adipocytes.

## 6. Conclusion

Accumulation of senescence cells is a hallmark of aging. Moreover, it is known that accumulation of these cells is increased in obesity. Although arrested in their cell cycle, these cells stay metabolically active and develop SASP, including secretion of cytokines thus leading to low-grade inflammation. Chronic low-grade inflammation which can be found in obesity leads to different disorders, like insulin resistance, diabetes, and hypertension. The present study demonstrates an anti-SASP effect of the selected polyphenols as well as spermidine, butyrate, and BHB on senescence induced preadipocytes. Additionally, resveratrol, anthocyanidin, and EGCG induced *SIRT3/NRF2*, a pathway believed to be reduced senescence.

## Data Availability

Data will be made available in a special file on request.
